# Constrained incremental tree building: new absolute fast converging phylogeny estimation methods with improved scalability and accuracy

**DOI:** 10.1186/s13015-019-0136-9

**Published:** 2019-02-06

**Authors:** Qiuyi Zhang, Satish Rao, Tandy Warnow

**Affiliations:** 1 Department of Mathematics, University of California Berkeley, Evans Hall, Berkeley, CA 94720 USA; 2 Department of Computer Science, University of California Berkeley, SODA Hall, Berkeley, CA 94720 USA; 3 Department of Computer Science, University of Illinois Urbana-Champaign, 201 N. Goodwin Avenue, Urbana, IL 61801 USA

**Keywords:** Phylogeny estimation, Short quartets, Sample complexity, Absolute fast converging methods, Neighbor joining, Maximum likelihood

## Abstract

**Background:**

Absolute fast converging (AFC) phylogeny estimation methods are ones that have been proven to recover the true tree with high probability given sequences whose lengths are polynomial in the number of number of leaves in the tree (once the shortest and longest branch weights are fixed). While there has been a large literature on AFC methods, the best in terms of empirical performance was $DCM_{NJ},$ published in SODA 2001. The main empirical advantage of ${DCM}_{NJ}$ over other AFC methods is its use of neighbor joining (*NJ*) to construct trees on smaller taxon subsets, which are then combined into a tree on the full set of species using a supertree method; in contrast, the other AFC methods in essence depend on quartet trees that are computed independently of each other, which reduces accuracy compared to neighbor joining. However, ${DCM}_{NJ}$ is unlikely to scale to large datasets due to its reliance on supertree methods, as no current supertree methods are able to scale to large datasets with high accuracy.

**Results:**

In this study we present a new approach to large-scale phylogeny estimation that shares some of the features of ${DCM}_{NJ}$ but bypasses the use of supertree methods. We prove that this new approach is AFC and uses polynomial time and space. Furthermore, we describe variations on this basic approach that can be used with leaf-disjoint constraint trees (computed using methods such as maximum likelihood) to produce other methods that are likely to provide even better accuracy. Thus, we present a new generalizable technique for large-scale tree estimation that is designed to improve scalability for phylogeny estimation methods to ultra-large datasets, and that can be used in a variety of settings (including tree estimation from unaligned sequences, and species tree estimation from gene trees).

## Introduction

The inference of phylogenies from molecular sequence data is generally approached as a statistical estimation problem, in which a model tree (equipped with a model of sequence evolution) is assumed to have generated the observed data, and the properties of the statistical model are then used to infer the tree. Various statistical approaches can be applied for this estimation, including maximum likelihood, Bayesian techniques, and methods that operate by computing a distance matrix and then computing the tree from the distance matrix.

Many stochastic sequence evolution models have been developed, starting with the Cavender–Farris–Neyman [1, 2] symmetric two-state model (referred to henceforth as “CFN”) and including increasingly complex molecular sequence evolution models (with four states for DNA, 20 states for amino acids, and 64 states for codon sequences). However, typically the theory that can be established under the CFN model can also be established under the more complex molecular sequence evolution models used in phylogeny estimation.

Under standard sequence evolution models, many methods are known to be statistically consistent (meaning that they will provably converge to the true tree as the sequence lengths increase), including maximum likelihood [3] and many distance-based methods [4, 5]. However, with the increasing availability of sequence datasets (e.g., the SILVA database has several million RNA sequences [6]), the need for phylogeny estimation methods that can be highly accurate on ultra-large datasets has increased. Therefore, a key challenge is to have methods that can scale to large datasets while maintaining good accuracy. Hence, the most desirable methods are those that run in polynomial time and that recover the true tree with high probability from short sequences (i.e., sequences that do not have very many sites). In this respect, methods that are “absolute fast converging” [7–9] (i.e., methods that recover the true tree with high probability from polynomial length sequences) are the most promising.

There are several methods that have been established to be absolute fast converging (AFC) under the CFN model, including maximum likelihood [3] (if solved exactly) and various distance-based methods [7–14]. Some of these algorithms achieve a poly-logarithmic sample complexity but require a balanced model tree and an upper bound on *g*, the maximum edge weight (defining the expected number of changes of a random site) in the CFN model. Specifically, methods based on reconstruction of ancestral sequences provide the best sample complexity bounds but cannot handle the case where *g* is larger than what is known as the Kesten–Stigum threshold, which is $\ln (\sqrt{2})$ [15] for the CFN model. The “short quartet” methods were the earliest AFC methods (which are AFC in the regime where *g* is unbounded), but these are designed to either return the true tree or else fail to return anything [7–9].

Two of the fastest AFC methods are the Harmonic Greedy Triplets (HGT + FP) method by Csűrös [16] and a method developed by King et al. [17]; these methods use $O(n^2)$ time and are based on quartet trees, and have the desirable property that they always return a tree for every input. Another good approach is $\texttt {DCM}_{NJ},$ which uses a divide-and-conquer technique [9]. In the first phase, $O(n^2)$ trees are computed, each based on dividing the sequence dataset into overlapping subsets, constructing trees on each subset using the polynomial time distance-based method neighbor joining (NJ) [18], and then combining the subset trees using a supertree method. This approach differs from other AFC methods in its use of neighbor joining to construct trees on subsets, whereas the other AFC methods in essence construct the tree by independently constructing quartet trees, and then assembling the quartet trees together using a quartet amalgamation method.

Very few AFC methods have been implemented; however, a study [10] comparing $\texttt {HGT}+\texttt {FP}$ [16] and $\texttt {DCM}_{NJ}$ [9] showed that $\texttt {DCM}_{NJ}$ had better accuracy. Since the theory does not predict this, the results on simulated data suggest that the use of $\texttt {NJ}$ to construct trees on subsets and then combine the trees using a supertree method may be empirically advantageous compared to methods that combine quartet trees that are estimated independently. Unfortunately, the reliance on a supertree method to combine subset trees means that $\texttt {DCM}_{NJ}$ is unlikely to scale to ultra-large datasets, because no current supertree method has shown the ability to maintain good accuracy and reasonable running times on large datasets [19].

The purpose of this paper is to describe a new polynomial time AFC phylogeny estimation method that should improve on $\texttt {DCM}_{NJ}{:}$ it is designed to have comparable accuracy to $\texttt {DCM}_{NJ}$ but also to be able to analyze ultra-large datasets (i.e., more than 100,000 sequences). The basic approach of this new method is similar to $\texttt {DCM}_{NJ}$ in that it uses divide-and-conquer, applies neighbor joining to subsets of the sequence dataset, and then merges the subtrees together. However, it differs from $\texttt {DCM}_{NJ}$ in a few important ways, which we describe below. Most importantly, it divides the taxon set into disjoint subsets and then merges the subset trees without relying on any supertree method; thus, it avoids the challenge of relying on existing supertree methods, none of which are likely to scale to large datasets. We present the results here initially for the CFN model, and then extend the results for the Generalized Time Reversible (GTR) model [20]. Our arguments for correctness are very similar to those in [16, 17].

## Background material

### Absolute fast convergence under the CFN model

Under the Cavender–Farris–Neyman (CFN) model, we have a rooted binary tree *T* and substitution probabilities *p*(*e*) on the edges *e* of *T*. The state at the root is 0 or 1 with equal probability, and the state changes on edge *e* with probability *p*(*e*), with $0< p(e) < 0.5$ for all edges *e*. This model can be used for sequence evolution by requiring that all the sites evolve *i.i.d.* down the tree. Finally, we define $w(e) = -\frac{1}{2}\log (1-2p(e)).$ We also define $CFN_{f,g}$ to be the set of all CFN model trees $(T,\Theta )$ (where $\Theta$ denotes the set of numeric parameters on the edges) with $f \le w(e) \le g$ for all edges in *T*, for arbitrarily selected positive real numbers $f \le g.$

#### **Definition 1**

A phylogeny estimation method $\Phi$ is said to be absolute fast converging (AFC) under the CFN model if, for all positive values $f,g,\epsilon$ (with $f \le g$), there is a polynomial *p* such that for all CFN model trees $(T,\Theta )$ in $CFN_{f,g}$ the method $\Phi$ will recover the unrooted tree topology *T* given sequences of length *p*(*n*) with probability at least $1 - \epsilon.$ Note that the polynomial will in general depend on *f*, *g* and $\epsilon$.

### DCM$_{NJ}$ + SQS: an AFC method with good empirical performance but low scalability

Here we describe the approach used in [9] called “DCM$_{NJ}$ + SQS”, which was shown to have high accuracy in simulation studies with up to 1600 sequences [10]. (Note: “DCM” refers to “disk-covering method” and “SQS” refers to the “short quartet support” criterion; see [5, 9]). The input is a set of sequences generated by an unknown model tree and a dissimilarity matrix *d* (i.e., a symmetric matrix that is zero on the diagonal) where $d_{ij}$ is the estimated distance between taxa $s_i$ and $s_j,$ based on the selected sequence evolution model. For example, when the model is CFN, then $d_{ij} = - \frac{1}{2}\ln (1- 2 H_{ij})$ will be the “empirical CFN distance”, where $H_{ij}$ is the Hamming distance between sequences *i* and *j* divided by the sequence length (i.e., the normalized Hamming distances). Note that these empirical CFN distances converge in probability to $D_{ij} = -\frac{1}{2}\log ( 1- 2E_{ij})$ where $E_{ij}$ is the expected normalized Hamming distance between sequences in leaves *i* and *j*, and that *D* is an additive matrix for the model tree. $\texttt {DCM}_{NJ}+\texttt {SQS}$ uses a two-phase structure, as follows.

- Phase 1: A set $\mathcal {T}$ of $O(n^2)$ trees is computed, with at most one tree $t_q$ for each entry *q* in the dissimilarity matrix *d*.
- Phase 2: All the trees in $\mathcal {T}$ are scored using the *SQS* criterion (where “SQS” refers to the short quartet support, defined in [9]) and the best-scoring tree is returned.

Before we can describe these phases, we need to provide some definitions.

#### **Definition 2**

(*From* [21]) For the given dissimilarity matrix *d* and positive real number *q*, we define the threshold graph *TG*(*d*, *q*) to be the graph with the *n* taxa as the vertex set and edges (*i*, *j*) if and only if $d_{ij} \le q.$ We also assign weight $d_{ij}$ to each edge (*i*, *j*) in *TG*(*d*, *q*). Hence, $TG(d,\infty )$ denotes the complete graph with edge weights given by the dissimilarity matrix *d*.

We use a standard technique, called the Four Point Method, to compute quartet trees (i.e., unrooted binary trees on four leaves) that is based on the Four Point Condition [22].

#### **Definition 3**

(*From* [7]) Given a four-taxon set $\{u,v,w,z\}$ and a dissimilarity matrix *d*, the Four Point Method ($\texttt {FPM}$) infers tree *uv*|*wz* (meaning the quartet tree with an edge separating *u*, *v* from *w*, *z*) if $d(u,v) + d(w,z) \le \min \{ d(u,w) + d(v,z), d(u,z) + d(v,w)\}.$ If equality holds, then the $\texttt {FPM}$ infers an arbitrary topology.

Phase 1 for $\texttt {DCM}_{NJ}+\texttt {SQS}$ is performed as follows. Given *q* (the selected threshold), the threshold graph *TG*(*d*, *q*) is computed, then edges are added to the graph to make it triangulated (if necessary), where a triangulated graph is one that has no simple cycles of size four or more; furthermore, if *d* is additive, then *TG*(*d*, *q*) is triangulated. Once the triangulated graph is computed, the set of all maximal cliques can be extracted in polynomial time. See [9] for additional details and proofs.

Trees are computed for each maximal clique using neighbor joining [18], and the trees on these cliques are then combined into a tree on the full set of species using a selected supertree method. Phase 2 uses the *SQS* criterion, but other criteria also have good theoretical properties. The *SQS* score of a tree *T*, defined by *SQS*(*T*), is the maximum *l* such that for all quartets $\{u,v,w,x\}$ of taxa with maximum interleaf distance at most *l*, the Four Point Method on $\{u,v,w,x\}$ produces a four-leaf tree that agrees with the *T*.

#### **Theorem 1**

(From [9, 10]) *The short quartet support (SQS) criterion score can be calculated for each tree* $t_q \in \mathcal {T}$ *in polynomial time. Also, there is a polynomial* *p(n)* *such that if* $\mathcal {T}$ *contains the true tree* *T* *and the sequences are of length* *p(n)* *(where* *n* *is the number of leaves), then with probability at least* $1-\epsilon,$ *the tree in* $\mathcal {T}$ *with the highest SQS score is the true tree* *T*.

The basic approach has good theoretical guarantees (i.e., it is AFC, and more generally if $\texttt {X}$ is any exponentially converging base method (meaning $\texttt {X}$ recovers the tree with high probability given exponentially many sites) and $\texttt {Y}$ is any true tree selection criteria that has the same theoretical properties as SQS (as given in Theorem 1), then $\texttt {DCM}_{X}+\texttt {Y}$ is AFC. Empirical performance of $\texttt {DCM}_{NJ}+\texttt {SQS}$ on simulated data was excellent, substantially outperforming $\texttt {NJ}$ and fast triplet/quartet-based greedy tree growing methods, such as $\texttt {HTP} +\texttt {FP},$ on large datasets especially when there was a high rate of evolution [10]. However, these studies were limited to datasets with at most 1600 sequences. In other words, $\texttt {DCM}_{NJ}+\texttt {SQS}$ was not tested on very large datasets, which is to some extent the point of absolute fast converging methods.

Furthermore, the design of $\texttt {DCM}_{NJ}$ suggests some limitations in terms of scalability to large datasets. Most importantly, supertree methods do not have good scalability, as all current supertree methods with good accuracy are attempts to solve NP-hard optimization problems, and so become computationally intensive on large datasets [19]. Hence, any reasonably fast method will need to completely avoid the supertree calculation step.

## Incremental tree-building ($\texttt {INC}$)

We begin by describing the $\texttt {INC}$ method in the unconstrained condition (i.e., when there are no constraint trees), and prove that it is AFC.

### High-level description of $\texttt {INC}$

The input to $\texttt {INC}$ is a dissimilarity matrix *d*. We present a high-level description of how tree $t = \texttt {INC}(d)$ is computed.

- Find the insertion ordering $\sigma = x_1, x_2, \ldots , x_n.$
- Initialize *t* as the three-leaf tree on the first three taxa in the ordering.
- For $i=4$ up to *n*.
  - Determine the set of valid quartets to use for placing $x_i$ into tree *t*.
  - Compute quartet trees for each valid quartet, and let them vote for where to place $x_i.$
  - Pick the edge *e* in *t* that has the most votes; if this is a tie, pick an edge at random from the set of edges with the most votes.
  - Insert $x_i$ into edge *e*.
- Return the resulting tree *t*.

Thus, at a high-level, the $\texttt {INC}$ algorithm operates by greedily growing a tree *t* based on a computed sequence addition ordering. Yet, many of the details of the algorithm are unspecified (e.g., we do not say how we calculate the insertion ordering, how we determine the set of valid quartets, how we compute trees on valid quartets, and how the quartet trees vote). Below, we provide details for each of the steps for this algorithm.

#### Computing the sequence addition ordering

**Fig. 1:**
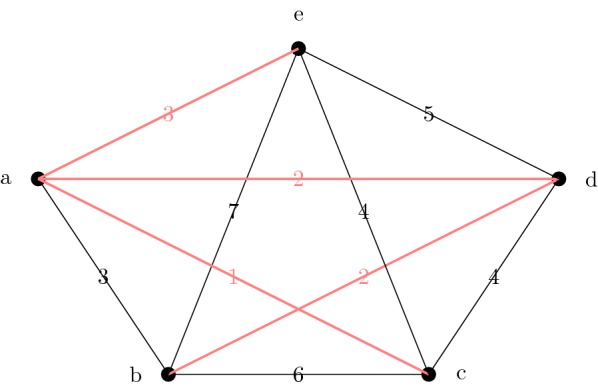
How the insertion ordering is computed. We show the threshold graph $TG(D,\infty )$ and a minimum spanning tree *S* in red. A possible insertion ordering produced by a BFS on *S* starting at *e* is *e*, *a*, *c*, *d*, *b*

We begin by constructing a minimum spanning tree *S* of $TG(d,\infty ),$ where *d* is the input dissimilarity matrix. Once the spanning tree *S* is computed, we choose an arbitrary leaf in *S* to be the starting vertex in the ordering. Then we order the vertices according to order of traversal in a BFS (or DFS) from the starting vertex. See Fig. 1 for an example.

#### How we compute the growing tree t

We initialize our tree *t* by choosing the first three taxa according to our insertion ordering and forming the three-leaf tree with an internal node that connects to all three taxa. In order to define how we insert the remaining taxa into *t*, we need to formally define the “valid quartets” and how we compute quartet trees for valid quartets.

##### **Definition 4**

Let $q_0$ be the maximum weight of an edge in *S* and let $q = 8q_0,$ A quartet of leaves is valid if its maximum pairwise distance (i.e., diameter) is at most *q*. The quartet tree for the valid quartet is computed using the Four Point Method.

As we will show later, the restriction to just the “valid quartets” allows us to develop a tree construction method that runs in polynomial time and that is AFC. Note also that restricting the diameter of the valid quartets to small values has a mixed effect: if the maximum permitted diameter is too small then even correct quartet trees will not be sufficient to reconstruct the tree, but if the maximum permitted diameter is too large then some computed quartet trees are more likely to be incorrect. We show that this setting for the maximum diameter *q* to be $8q_0$ is sufficient to allow us to prove that the algorithm is AFC. However, we did not try to optimize this constant, and hence the choice of the constant 8 is likely not optimal (i.e., smaller constants might give better theoretical results).

There may be many valid quartets, but we only need to examine a linear number of these, as we now show. Suppose we wish to add a vertex *x* into *t*. Given an internal node *u* of *t*, because *t* is binary the removal of *u* splits *t* into three non-empty components which we will refer to as $t_1,t_2,t_3$ (with internal nodes included). Because the leaves of *t* form a connected induced subtree of *S*, we can find taxa $u_i$ in $t_i$ and $v_i \in V(t)\setminus t_i$ such that $(u_i,v_i)$ is an edge in *S*, for $i=1,2,3.$ We will associate the triplet $u_1,u_2,u_3$ to node *u*, so that the addition of leaf *x* defines a quartet, and we can check to see if the quartet is valid. We summarize this discussion with the following definition.

##### **Definition 5**

Let *u* be an internal node of a binary tree *t* and let $u_1,u_2,u_3$ be leaves in the three components of *t* upon removing *u* such that there exists $v_i$ with $(u_i, v_i)$ an edge in *S* where $v_i$ is not in the same component as $u_i.$ Then, for some choice of $q \in \mathbb {R}^+,$ a quartet query on $\{u_1,u_2,u_3,x\}$ is *q*-valid iff the *d*-diameter (maximum pairwise distance with respect to the input matrix *d*) of $\{u_1,u_2,u_3,x\}$ is less than *q*. We use the term valid when *q* is clear from context.

**Fig. 2:**
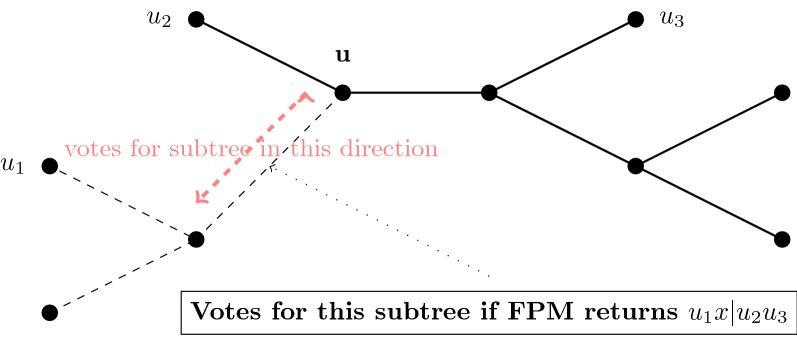
How quartet queries vote. Each node *u* in the tree *t* is associated with a triplet $u_1, u_2, u_3$ from each of the three subtrees around *u* (see text for details). When adding new taxon *x*, we make quartet queries, such as the one on $\{u_1, u_2, u_3, x\},$ which potentially contribute to the vote for where to place *x*. Only those quartets whose diameter is below a specified threshold are allowed to vote, and these are referred to as “valid quartets”. Trees are computed on these valid quartets using the Four Point Method. Each valid quartet query then votes using the corresponding quartet tree by identifying a subset of the tree in to which *x* can be placed without violating the quartet tree. In this figure, FPM returns quartet tree $u_1x|u_2u_3,$ and then votes for all the edges in the subtree off *u* containing $u_1$ (edges dashed). With high probability, given polynomial sequence lengths, valid queries make correct votes

To determine how to place leaf *x* into the growing tree *t*, we compute a tree for each valid quartet query (which includes *x*) using the Four Point Method. For example, if the tree computed on valid quartet query $u_1,u_2,u_3,x$ (with $u_i \in t_i,$ and $t_1,t_2,t_3$ the three subtrees off internal node *u*) returns $xu_1|u_2 u_3,$ then this implies that *x* should be placed in the subtree of *t* induced on $t_1 \cup {u}$ hence, each edge in that subtree will receive a vote (Fig. 2).

**Fig. 3:**
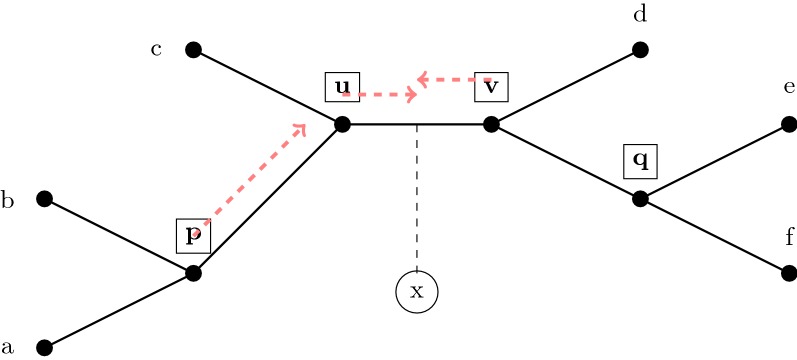
Using quartet queries to place new taxa. When adding a taxon *x* into the tree t, all valid quartet queries are allowed to vote for the edges in the tree (see Fig. 2) and *x* is then added to the edge that receives the most votes. In this figure, we show *x* being placed into edge *uv*, based on the following possible vote outcomes: The query at *p* returns tree *ab*|*xc* and so votes for $\{pu, uc, uv, vd, vq, qe, qf\},$ and similarly the queries at *u* and *v* vote for $\{uv, vd, vq, qe, qf\}$ and $\{uv, uc, up, pb, pa\},$ respectively. The query at *q* is not valid and does not get to vote. Our algorithm guarantees that queries at *u*, *v* are valid with high probability. Note that internal nodes are boxed and taxon nodes are circled

Thus, each valid quartet adds a vote to each edge in some non-empty subtree of the tree. We define the support of an edge in the tree *t* to be the number of valid quartet trees that voted for that edge. We then choose an edge *e* in the tree *t* that has the largest total support (and if there is a tie, we pick an edge *e* at random with the maximum support). We then subdivide *e* and then make *x* adjacent to the node created. See Fig. 3 for an illustration of how this voting procedure operates.

Furthermore, as we will shortly show, when the sequences are long enough, then there will be a unique edge on which all queries agree, and so the algorithm will correctly add *x* into *t* (in such a way that it agrees with the model tree *T*), and so inductively the greedy algorithm will construct the model tree.

### Theoretical properties of $\texttt {INC}$ under the CFN model

We now establish the theoretical properties of $\texttt {AFC}$ under the CFN model. Throughout this section, recall that $d_{ij}$ is the empirical CFN dissimilarity between taxa *i*, *j* and $D_{ij}$ is the underlying CFN model distance between taxa *i*, *j* defined by the model tree $(T,\Theta )$ with edge weights given by $w(e)=-\frac{1}{2}\log (1-2p(e)).$ Given matrices *d* and *D* and positive real *q*, we define $\epsilon (q) = \max \{|d_{ij}-D_{ij}|: d_{ij} \le q \text { or } D_{ij} \le q\}.$

#### **Theorem 2**

*Let* *d* *be a dissimilarity matrix*, *D* *an additive matrix defining a model tree* *T* *with edge weights* $w: E(T) \rightarrow \mathbb {R}^+,$ *and* $q = 8q_0,$ *where* $q_0$ *is the maximum distance in the minimum spanning tree of* $TG(d,\infty ).$ *Let* *f* *be the weight of the shortest internal edge in* *T* *and suppose* $\epsilon (q) < f/2$ *and* $q_0 \ge f/2,$ *then* INC*(d)* *returns* *T*.

#### *Proof*

We proceed by induction on the number of leaves in *T*. Our claim holds when *T* has three leaves, since there is only one such topology. Let *t* be the tree maintained by INC before the insertion of the last taxon *x* according to our insertion ordering. By induction, *t* must have the correct topology. It suffices to show that valid queries can determine the accurate placing of *x*. Assume that the correct location on which to place *x* is $e = (u,v)$ of *t*. Note that when $\epsilon (q) < f/2,$ the Four Point Method is guaranteed to correctly construct quartet trees for all quartets with diameter at most *q* (and hence for all valid quartets), so that all valid node queries will return the correct quartet tree. Therefore, *e* will receive all possible votes.

To show that all other edges will miss at least one vote, it suffices to show that node queries are valid at *u* and *v* because all other edges will miss a vote from one of the two such queries, confirming that *x* is inserted at *e* (if one of *u*, *v* is a leaf vertex, the same conclusion is achieved).

We will show that a node query at *u* is valid; a similar argument is done for *v*. Let $u_1, u_2, u_3$ be selected upon the deletion of *u* with their corresponding $v_i.$ First, for all *i*, $D(u_i,v_i) \le q_0 + f/2$ since $d(u_i,v_i)\le q_0$ and $\epsilon (q) \le f/2.$ Then, since $v_i \in V\setminus t_i$ and *t* is the correct tree topology, $D(u_i,u) \le D(u_i,v_i) \le q_0 + f/2,$ where *D* is the true distance matrix and *u* is the corresponding internal node in *T*.

Next, we claim that $D(x,u) \le 2q_0+f$ and together we will have concluded that $\{u_1,u_2,u_3,x\}$ is a quartet of *d*-diameter at most $3q_0+2f$ since by triangle inequality, we deduce $\{u_1,u_2,u_3,x\}$ is of *D*-diameter $3q_0+3f/2$ and so the *d*-diameter is bounded at $3q_0+2f \le 7q_0 \le q,$ proving validity.

For the last claim, since *x* is within $q_0$ of some leaf $x'$ in *t*, $D(x,x') \le d(x,x') + f/2 \le q_0 + f/2.$ Since the path *x* to $x'$ must pass through *u* or *v* in *T* and $e = (u,v)$ is the correct edge insertion location, we conclude that $\min (D(x,u),D(x,v)) \le q_0 + f/2.$

If $D(x,u) \le q_0+f/2,$ then our claim follows (we automatically get $D(x,u) \le 2q_0 + f$). Else, $D(x,v) \le q_0+f/2.$ Since $v \in V(t_3),$ $D(x,u) \le D(x,v) + D(u,v) \le q_0+f/2 +D(u,v) \le q_0 +f/2 + D(u,u_3) \le q_0 +f/2 + q_0 + f/2 \le 2q_0+f.$

Therefore, if the correct edge is (*u*, *v*), then the query at *u* votes against all edge placements for *x* in $t_1 \cup t_2,$ and symmetrically for *v*. Hence all other edges will miss at least one vote. $\square$

#### **Theorem 3**

*Given the* $n \times n$ *dissimilarity matrix* *d* *and a given* *q,* $\texttt {INC}(d)$ *can be implemented in* $O(n^2)$ *time and space.*

#### *Proof*

It suffices to show that as we grow our tree *t*, each insertion step can be implemented to take *O*(*n*) time. First, each internal node, when initialized due to an inserted taxon, can store the necessary vertices $u_1,u_2,u_3$ and that can be done in *O*(*n*) time. Each node query is *O*(1), so in total the |*V*(*t*)| node queries take *O*(*n*) time.

The only possible difficulty is efficiently finding the edge with the most votes. A naive implementation will lead to an $O(n^3)$ runtime. For any edge $e = (u,v)$ on the tree, let *n*(*e*) denote the total number of votes that *e* has. Note that if $e, e'$ are adjacent edges in *t* with common vertex *u*, then $n(e) - n(e')$ can be determined by simply looking at the short quartet query at *u*. Specifically, let $e = (u,v)$ and $e' = (u,v')$ be adjacent edges at vertex *u*. If the query at *u* was invalid or it showed that *x* should be in $t_j$ with $v,v' \not \in t_j,$ then $n(e) - n(e') = 0.$ Otherwise, if the query returned $t_j$ with $v \in t_j,$ then $n(e) - n(e') = 1;$ a similar argument shows that if $v' \in t_j$ then $n(e) - n(e') = -1.$ Therefore, by using this local property, we can calculate $n(e) + C$ for some constant *C* in *O*(*n*) time by performing $\texttt {BFS}$ starting from any leaf of the tree. The $\texttt {BFS}$ then simply returns the edge with the highest score.

Finally, throughout the algorithm, we need to store the dissimilarity matrix, spanning tree, insertion ordering, and *O*(1) vertices at each node of the tree. Together, this requires $O(n^2)$ space. $\square$

#### **Theorem 4**

(Azuma’s inequality [23]) *Suppose* $X = (X_1, X_2, ..., X_k)$ *are independent random variables taking values in any set S, and let* $L:S^k \rightarrow \mathbb {R}$ *be any function that satisfies the condition:* $|L(u)-L(v)| \le t$ *whenever u and v differ in just one coordinate. Then,*

$$\begin{aligned} P(|L(X) - E[L(X)]| \ge \lambda ) \le 2 \exp \left( -\frac{\lambda ^2}{2t^2k} \right) \end{aligned}$$

#### **Theorem 5**

*For* $k =\Omega \left( \frac{\ln (n/\epsilon )e^{4q}}{f^2} \right),$ *with probability* $\ge 1-\epsilon,$ *we have* $\epsilon (q) < f/2.$ *Furthermore, if* $q_0$ *is the minimum value of* *q* *such that* *TG*(*d*, *q*) *is connected, then* $q_0 = O(g\log n)$ *and* $q_0 \ge f/2.$

#### *Proof*

To show that $\epsilon (q) < f/2,$ we must show that $|D_{ij} - d_{ij}| < f/2$ when $D_{ij} < q$ or $d_{ij} < q.$ First, if $D_{ij} < q$ we show that $k =\Omega \left( \frac{\ln (n/\epsilon )e^{2q}}{f^2} \right)$ suffices to show $|D_{ij} - d_{ij}| < f/2$ holds with probability $\ge 1-\epsilon /n^2.$ We express our probability of failure as:

$$\begin{aligned} P(|D_{ij} - d_{ij}| \ge f/2)&= P\left( \left| \log \left( \frac{1 - 2H_{ij}}{1-2E_{ij}}\right) \right| \ge f \right) \\&\le P((1- 2 E_{ij})e^{-f} \ge 1 - 2 H_{ij}) + P(1 - 2 H_{ij} \ge (1-2E_{ij})e^f) \end{aligned}$$

The first expression can be written as:

$$\begin{aligned} P(H_{ij} - E_{ij} \ge \frac{1}{2}(1-e^{-f})(1-2E_{ij}))&\le P(|H_{ij} - E_{ij}| \ge \frac{1}{2}(1-e^{-f})e^{-2D_{ij}})\\&\le 2\exp (-\Omega (k(1-e^{-f})^2e^{-4q}))\\&\le 2 \exp (-\Omega ( \ln n/\epsilon )) \end{aligned}$$

The second line follows from Azuma’s (Theorem 4) with $t = 1/k.$ Similarly, the second expression is equivalent to:

$$\begin{aligned} P(E_{ij} - H_{ij} \ge \frac{1}{2}(e^{f}-1)(1-2E_{ij}))&\le P(|E_{ij} - H_{ij}| \ge \frac{1}{2}(e^{f}-1)e^{-2D_{ij}})\\&\le 2\exp (-\Omega (k(e^{f}-1)^2e^{-4q}))\\&\le 2 \exp (-\Omega ( \ln n/\epsilon )) \end{aligned}$$

Next, if $d_{ij} < q,$ then we show that $D_{ij} < 2q+1$ with high probability and then apply the previous result with $q' = 2q+1.$ First, if we let $r_{ij} = D_{ij} - q,$ then by simple algebra our probability that $d_{ij} < q$ when $r_{ij} > q+1$ is bounded by

$$\begin{aligned} P(d_{ij} < q )&= P(D_{ij} - d_{ij}> D_{ij}-q)\\&= P\left( -\frac{1}{2}\log \left( \frac{1-2E_{ij}}{1-2H_{ij}} \right)> D_{ij}-q \right) \\&= P(1-2H_{ij}> e^{2r_{ij}}(1-2E_{ij}))\\&= P\left( E_{ij} - H_{ij}> \frac{1}{2}(e^{2r_{ij}}-1)e^{-2D_{ij}}\right) \\&= P\left( |E_{ij} - H_{ij}| > \frac{1}{4}e^{2r_{ij}-2D_{ij}}\right) \\&\le 2\exp (-\Omega (ke^{-4q}))\\&\le 2 \exp (-\Omega ( \ln n/\epsilon )) \end{aligned}$$

The fourth to fifth line follows since $e^{2r_{ij}} - 1 > \frac{1}{2}e^{2r_{ij}}$ whenever $r_{ij} > 1.$ Therefore, by a union bound, we conclude our claim that $\epsilon (q) < f/2$ with probability $\ge 1-\epsilon.$

We now show that $q_0 = O(g\log n).$ Note that in our model tree, since *g* is the maximum weight of an edge in a binary tree with *n* leaves, it follows that $TG(D,O(g\log n))$ is connected. By our previous part, we know that $|d_{ij} - D_{ij}| < f/2$ for all edges in $TG(D,O(g\log n)).$ Therefore, we conclude that $TG(d, O(g\log n))$ is also connected, and so $q_0 = O(g\log n).$

Lastly, we show $q_0 \ge f/2.$ Consider all edges in the minimum spanning tree of $TG(d,\infty ).$ These edges have true weight (in *D*) at least *f* and by our previous part the weight of the edges in the minimum spanning tree deviate from the true weight (as defined by the model tree) by at most *f* / 2. Thus, we conclude that $q_0\ge f/2.$ $\square$

#### **Theorem 6**

$\texttt {INC}$ *is absolute fast converging for the CFN model and takes* $O(n^2)$ *time and space (assuming distances are precomputed).*

#### *Proof*

All we need to establish is that for every triplet $\epsilon ,f,g$ with $0<f<g<\infty$ and $\epsilon >0,$ there is a polynomial *p*(*n*) such that for all model trees $(T,\Theta )$ in $CFN_{f,g},$ given sequences of length at least *p*(*n*) generated by $(T,\Theta )$ and empirical CFN dissimilarity matrix *d* computed on these sequences, the tree returned by $\texttt {INC}(d)$ is the model tree *T* with probability at least $1-\epsilon.$

Let *q* be the maximum edge weight on the minimum spanning tree in the underlying model tree. We know $q = O(g \log n).$ By Theorem 5, with probability $\ge 1-\epsilon,$ when $k = \Omega (\ln (n/\epsilon )e^{4q}/f^2) = poly(n),$ we have $\epsilon (q) < f/2$ Furthermore, $q_0 \ge f/2.$ Therefore, by Theorem 2, the insertion algorithm will return the model tree *T*. Hence, $\texttt {INC}$ is absolute fast converging for the CFN model.

Finally, given the dissimilarity matrix, the running time and space complexity to compute and store the minimum spanning tree and to run INC are all $O(n^2)$ by Theorem 3.$\square$

The method we described runs in $O(n^2)$ time and is AFC. As shown by [17], any algorithm that reconstructs the true tree with high probability and uses distance calculations as its only source of information about the phylogeny on sequences of length $O(poly\log n)$ will have $\Omega (n^2)$ runtime, up to logarithmic factors. Hence, our runtime is optimal.

## Boosting $\texttt {INC}$ using constraint trees

We show how $\texttt {INC}$ can be modified to take an arbitrary set of disjoint constraint trees. We present several variants of this method: one that optimizes speed while still guaranteeing that the resultant algorithm is AFC, and other variants that are designed for improved empirical accuracy but with an increase in running time and potentially a loss of the AFC property.

### Constrained $\texttt {INC}$

The input to the constrained version of $\texttt {INC}$ includes a set of leaf-disjoint trees. Therefore, the constraint set is compatible (i.e., a compatibility tree exists). We will describe a straightforward modification to $\texttt {INC}$ so that it never violates the topological information in the constraint trees, by which we mean only that the final output tree is required to induce each constraint tree, when restricted to that specific set of leaves. Thus, the constraint trees are not required to be clades in the output tree.

**Fig. 4:**
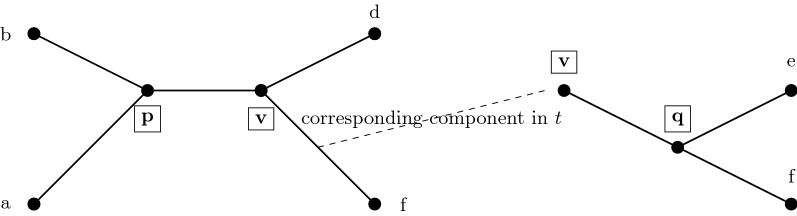
How constrained INC uses constraint trees to insert taxa. Left: the induced tree topology on taxon set $\{a,b,d,f\}$ of *t* in Fig. 3. Right: the subtree of *t* (on leaf set $\{v,q,e,f\}$ corresponding to the edge *vf* from the left subfigure

Before proceeding, we define the induced tree topology, $t^{A},$ on a tree *t* and a subset of leaves *A* as follows. Consider the minimal subtree of *t* that contains the leaves *A*, and then suppress all nodes of degree two (i.e., replace all maximal paths of degree two nodes by a single edge). The endpoints of an edge $e \in t^{A}$ correspond to vertices in *t* and so *e* corresponds to a (possibly length 1) path in *t*; we denote the corresponding endpoints of *e* as $e_t(u)$ and $e_t(v).$ For an induced tree $t^{A},$ the component in *t* corresponding to an edge $e \in t^A$ is the set of edges and vertices that can be reached by a walk starting from $e_t(u)$ and ending at $e_t(v)$ without using $e_t(u)$ or $e_t(v)$ multiple times. Note this will be a subgraph of *t* that includes $e_t(u)$ and $e_t(v)$ as leaves. See Fig. 4.

When working with constraint trees, we alter the insertion algorithm to account for the constraints. Recall that the constraint trees are on disjoint sets of taxa. Thus, when inserting a taxon, we identify the corresponding constraint tree $t_c$ that includes that new taxon. Let *A* be the set of taxa shared between $t_c$ and *t* and note that $t_c^{A} = t^{A}$ since the trees are consistent. Thus, in $t_c^{A\cup \{x\}},$ the taxon *x* can be viewed as being attached to some edge *e* in $t^{A}.$ The eligible edges are in the component in *t* corresponding to *e*. We then simply run $\texttt {INC}$ on the component to find the desired edge of insertion.

### $\texttt {INC}{-}\texttt {NJ}$

We continue by presenting the $O(n^2)$ $\texttt {INC}{-}\texttt {NJ}$ method, which is AFC and uses neighbor joining on carefully selected subsets to achieve this running time. Given the selected threshold *q*, we take our threshold graph *TG*(*d*, *q*) and compute a greedy disjoint clique decomposition, such that each clique is of size $O(\sqrt{n}).$ This is done by a simple ball-growing procedure that is interrupted when the ball includes more than $O(\sqrt{n})$ vertices; it is easy to see this takes $O(n^2)$ time. Next, we run $\texttt {NJ}$ on each of these clique; this produces a set of neighbor joining subset trees that will serve as our constraint trees. Since the subsets are disjoint, the subset trees are guaranteed to be compatible (meaning that a compatibility supertree exists). Finally, if $\epsilon (q) < f/2,$ then the following theorem guarantees that all constraint trees produced by our technique are correct. Furthermore, since $\texttt {NJ}$ applied to each clique of size $O(\sqrt{n})$ takes at most $O(n^{1.5})$ time, our total runtime over all cliques is $O(n^2).$

#### **Theorem 7**

*Neighbor Joining* *(*$\texttt {NJ}$*)* *will recover the true tree* *T* *whenever the input matrix* **d** *satisfies* $L_{\infty }(d,D) < f/2,$ *where* *D* *is an additive matrix defining an edge-weighted version of the true tree* *T* *and* *f* *is the shortest internal branch weight in* *T*. *Furthermore, the runtime and space complexity is* $O(n^3).$

#### *Proof*

The first sentence follows from [4]. The second sentence follows easily from the description of the algorithm.

#### **Theorem 8**

$\texttt {INC}{-}\texttt {NJ}$ *is AFC and has runtime and space complexity* $O(n^2)$ *(assuming distances are precomputed).*

#### *Proof*

Follows directly from combining Theorems 6 and 7. $\square$

### Boosting $\texttt {INC}$ using other constraint trees

The version of constrained-$\texttt {INC}$ we presented in the previous section achieves an optimal running time, but is based on obtaining constraint trees using neighbor joining on small subsets. However, we could modify the decomposition to produce larger subsets and thus take advantage of the likely improvement in accuracy obtained by using neighbor joining on these larger subsets to produce the constraint trees. This would still produce a polynomial time algorithm, but one with a higher (and hence suboptimal) running time.

Another variation would be to use other phylogeny estimation methods besides neighbor joining. In particular, maximum likelihood could be used to construct the constraint trees. As shown in [3], maximum likelihood under the CFN model is AFC. Hence, running maximum likelihood on subsets of the taxon set produced using the same technique as for $\texttt {INC}{-}\texttt {NJ}$ will provide correct subset trees from polynomial length sequences with high probability. Hence, the same approach we describe for use with neighbor joining (where that subset trees are computed using neighbor joining) can be modified to be used with maximum likelihood, and will be an AFC method. One negative impact of doing this would be running time, since maximum likelihood is NP-hard [24] and heuristics for maximum likelihood are computationally more intensive than neighbor joining.

## Extension to the generalized time reversible (GTR) Markov models

In this section, we extend our AFC convergence guarantees to the generalized time reversible (GTR) Markov model [20], which is the most commonly used site evolution model used in phylogenetics. We will show that $\texttt {INC}$ is AFC for the GTR model, drawing on established techniques and theory which we now present and summarize.

The GTR model can be seen as a special case of the general Markov model [25], which we first describe. Under the general Markov model on $m \ge 2$ states, we have a rooted binary tree *T* with some distribution of states $\pi > 0$ at the root of the tree and *m*-by-*m* stochastic transition matrices *M*(*e*) for each edge *e* of *T*. Starting with a random sequence drawn i.i.d. from $\pi,$ each site of the sequence evolves i.i.d. down the tree, according to the transition matrices specified by the model.

### **Definition 6**

(*GTR model*) Let $\Phi$ be the set of states with $|\Phi | = m.$ Let *Q* be an $m \times m$ rate matrix with $Q_{ij} > 0$ for $i \ne j$ and $\sum _{j \in \Phi } Q_{ij} = 0$ for all $i \in \Phi.$ Assume that *Q* satisfies detailed balance with respect to $\pi,$ or $\pi _i Q_{ij} = \pi _j Q_{ji}.$ Then, the General Time Reversible model is a general Markov model on $\Phi$ with $\pi$ as the initial state distribution and $M(e) = e^{\tau _e Q}$ for some $\tau _e > 0$ for each edge.

Note that the GTR model captures the CFN model by setting $m =2,$ $\pi = (1/2, 1/2)$ and

$$\begin{aligned} Q = \begin{bmatrix} -1/2&1/2 \\ 1/2&-1/2 \end{bmatrix} \end{aligned}$$

If we just consider one site, let $f_{ij}(\alpha ,\beta )$ be the probability that leaf *i* is in state $\alpha$ and leaf *j* is in state $\beta.$ By some indexing of the states, we can form a *m*-by-*m* square matrix $F_{ij} = [f_{i,j}(\alpha ,\beta )].$ Then, we define the true distance function to be $D_{ij} = - \log \det ( F_{ij}).$ Let $\hat{f}_{ij}(\alpha ,\beta )$ be the empirical probability (or relative frequency) that leaf *i* and leaf *j* are in state $\alpha , \beta$ respectively, calculated by taking a simple average over all *k* sequence sites. Similarly, we define $\hat{F}_{ij} = [\hat{f}_{i,j}(\alpha ,\beta )]$ and the empirical distance function to be $d_{ij} = - \log \det ( \hat{F}_{ij}).$ This log-det distance function is well-known for the general Markov model (and hence applicable for its submodels, including the GTR model) and is a generalization of the CFN distance function that also satisfies similar properties [25]. Note that the empirical distance function will converge to the true distance function as the number of sites $k \rightarrow \infty.$ With this extended definition of $d_{ij}, D_{ij},$ we define $\epsilon (q)$ similarly.

Note that $\det (M(e))$ takes the values 1 or – 1 precisely if *M*(*e*) is a permutation matrix. Also, for the CFN model, $det(M(e)) = 1 - 2p(e),$ where *p*(*e*) is the substitution probability on edge e; thus we know that $\det (M(e))>0$ and $\det (M(e)) \rightarrow 0$ as $p(e) \rightarrow 0.5,$ which correctly supports the intuition that information loss is large when *p*(*e*) is close to 0.5. In general, $(1/2)[1 -\det (M(e))]$ plays the role of *p*(*e*) in the general model and $-\log (\det (M(e)))$ is the corresponding *w*(*e*).

Thus, the natural extension of $CFN_{f,g}$ model to the GTR model, $GTR_{f,g},$ is to enforce $f \le -\log (\det (M(e))) \le g$ for all edges in all model trees in $GM_{f,g}.$

### **Definition 7**

A phylogeny estimation method $\Phi$ is said to be absolute fast converging (AFC) under the GTR model if, for all positive values $f,g,\epsilon$ (with $f \le g$), there is a polynomial *p* such that for all GTR model trees *T* in $GTR_{f,g},$ the method $\Phi$ will recover *T* given sequences of length $k = O(p(n))$ with probability at least $1 - \epsilon.$

Now, we apply INC with the extended version of the empirical distance matrix and we define *TG*(*d*, *q*), $q_0,$ $\epsilon (q)$ analogously for our generalized distance measure. Note that by our Markov property and time-reversibility, if *P* is the unique path between leaves *i*, *j* in *T*, then the true distance matrix is additive and satisfies $D_{ij} = \sum _{e \in P} - \log (\det (M(e)))$ up to a constant shift [8]. Thus, if $\epsilon (q) < f/2,$ then applying a quartet query via the Four Point Method will return the accurate quartet tree. Therefore, the claim that $\texttt {INC}$ is AFC for the GTR model follows immediately after we establish a slightly extended concentration bound.

### **Theorem 9**

*For* $k =\Omega (\frac{\ln (n/\epsilon )e^{4q}}{f^2}),$ *with probability* $\ge 1-\epsilon,$ *we have* $\epsilon (q) < f/2$ *for all* $q = O(g \log n)$ *in the GTR model.* *Furthermore, if* $q_0$ *is the minimum value of* *q* *such that* *TG(d,* *q)* *is connected, then* $q_0 = O(g\log n)$ *and* $q_0 \ge f/2.$

### *Proof*

Since the log-det distance function is still additive and corresponds to a tree metric with *f*, *g* still as minimum and maximum distances of an edge in the tree metric, our proof for the GTR model follows analogously once the following concentration equality is proved:

$$\begin{aligned} P(|\det (\hat{F}_{ij}) - \det (F_{ij})| \ge x) \le e^{-\Omega (kx^2)} \end{aligned}$$

By Hadamard’s inequality, we note that

$$\begin{aligned} \left |\det (\hat{F}_{{ij}} ) - \det (F_{{ij}} ) \right| \le m^{{m + 1}} \left\| {\hat{F}_{{ij}} - F_{{ij}} } \right\|_{{\max }} \max \left( {\left\| {F_{{ij}} } \right\|_{{\max }} ,\left\| {\hat{F}_{{ij}} } \right\|_{{\max }} } \right) \end{aligned}$$

Since *m* is regarded as a constant (for DNA models, $m = 4$) and since $F_{ij}, \hat{F}_{ij}$ are matrices whose entries denote frequencies or probabilities and thus are all bounded by 1, we conclude that it suffices to prove:

$$\begin{aligned} P\left( {\left\| {\hat{F}_{{ij}} - F_{{ij}} } \right\|_{{\max }} \ge x} \right) \le e^{{ - \Omega (kx^{2} )}} \end{aligned}$$

For each of the $m^2$ entries of $\hat{F}_{ij}, F_{ij},$ which are $\hat{f}_{ij}(\alpha ,\beta ), f_{ij}(\alpha ,\beta )$ respectively for all pairs of states $\alpha ,\beta \in \Phi,$ we can prove concentration by applying Azuma’s inequality (Theorem 4) for sum of *k* indicators.

$$\begin{aligned} P\left( \left |\hat{f}_{ij}(\alpha ,\beta )- f_{ij}(\alpha ,\beta ) \right| \ge x \right) \le e^{-\Omega (kx^2)} \end{aligned}$$

And our conclusion follows. $\square$

### **Theorem 10**

$\texttt {INC}$ *is absolute fast converging for the GTR model and takes* $O(n^2)$ *time and space (assuming distances are precomputed).*

## Discussion

This paper presents a novel algorithmic technique for constructing phylogenetic trees, which allows statistically consistent and highly accurate methods to be used on subsets of the taxa in a divide-and-conquer framework. We proved that several of these variants are absolute fast converging (AFC) under the CFN and GTR sequence evolution models, and that some of these methods achieve $O(n^2)$ time and space (where *n* is the number of sequences) once the dissimilarity matrix relating the sequences is computed. More generally, tree estimation methods could be scaled to large datasets using this approach, provided that it is possible to compute a matrix of pairwise distances that is guaranteed to converge to an additive matrix as the amount of data increases.

Many of the ideas in the algorithmic design of $\texttt {INC}$ are derived from prior algorithms for similar problems. For example, the node query insertion procedure was explored earlier in [26, 27] to produce a fast $O(n \log n)$ tree-growing algorithm, but analyzed under a different model of quartet tree error than we use here; furthermore, a direct implementation of their model does not produce an AFC algorithm. The idea of using a distance matrix to combine disjoint trees was originally presented in [28] in the context of multi-locus species tree estimation; that technique (called NJMerge) is a modification of the neighbor joining method [18] to allow for constraint trees. Since neighbor joining is not AFC [29], it seems unlikely that NJMerge can be used to produce an AFC method.

The goal of this work was improved empirical performance, compared to prior AFC methods. One of the interesting aspects of $\texttt {INC}$ is the potential to use it with disjoint constraint trees. As noted in this paper, maximum likelihood (if solved exactly) is AFC under the standard sequence evolution models, and although it is NP-hard to solve exactly there are many seemingly good heuristics for maximum likelihood (e.g., RAxML [30]). One possible technique that could result in excellent empirical accuracy and scalability is to use a maximum likelihood heuristic to compute constraint trees, and then combine the constraint trees using constrained $\texttt {INC}.$ The value of such an approach, however, depends on the decomposition strategy, as not all decompositions will produce AFC methods. Consider, for example, the impact of selecting quartets from a large phylogeny: if these quartets are selected arbitrarily, it is possible to produce quartets whose true trees fall in the Felsenstein Zone, where methods such as maximum parsimony are not statistically consistent and maximum likelihood—although consistent—will tend to have poor accuracy unless very long sequences are available [31, 32]. Therefore, the use of constrained $\texttt {INC}$ with maximum likelihood, or any other tree estimation method, will only be AFC for *some* decomposition strategies (and not for arbitrary ones), and may not provide accuracy advantages except when combined with carefully designed decomposition strategies.

Thus, additional work is needed to develop provably AFC divide-and-conquer strategies that enable this kind of approach to be used to the greatest empirical advantage on large challenging phylogenetic datasets.
